# Crystal structure and Hirshfeld surface analysis of (*E*)-3-(benzyl­idene­amino)-5-phenyl­thia­zolidin-2-iminium bromide

**DOI:** 10.1107/S2056989020001899

**Published:** 2020-02-21

**Authors:** Gulnara Sh. Duruskari, Mehmet Akkurt, Gunay Z. Mammadova, Taras Chyrka, Abel M. Maharramov

**Affiliations:** a Organic Chemistry Department, Baku State University, Z. Xalilov str. 23, Az, 1148 Baku, Azerbaijan; b Department of Physics, Faculty of Sciences, Erciyes University, 38039 Kayseri, Turkey; c Department of Theoretical and Industrial Heat Engineering (TPT), National Technical University of Ukraine "Igor Sikorsky Kyiv Polytechnic Institute", 03056, Kyiv, Ukraine

**Keywords:** crystal structure, charge assisted hydrogen bonding, thia­zolidine ring, envelope conformation, Hirshfeld surface analysis

## Abstract

The central thia­zolidine ring of the title salt, C_16_H_16_N_3_S^+^·Br^−^, adopts an envelope conformation, with the C atom bearing the phenyl ring as the flap atom. In the crystal, the cations and anions are linked by N—H⋯Br hydrogen bonds, forming chains parallel to the *b*-axis direction. Hirshfeld surface analysis and two-dimensional fingerprint plots indicate that the most important contributions to the crystal packing are from H⋯H (46.4%), C⋯H/H⋯C (18.6%) and H⋯Br/Br⋯H (17.5%) inter­actions.

## Abstract 2

In the crystal, the cations and anions of the title salt are linked *via* N—H⋯Br hydrogen bonds. In the ^1^H NMR spectra of this compound, the NH iminium protons were observed at *δ* 10.35 p.p.m., which confirms the strong charge-assisted hydrogen bonding (CAHB) in the =HN^+^—H**⋯**Br^−^ synthon.

## Chemical context   

Sulfur and nitro­gen-containing heterocycles maintain their importance as key fragments of drugs and medicinally active compounds (Pathania *et al.*, 2019 ▸). Moreover, azomethine-containing structural motifs have been widely employed for industrial purposes as they exhibit a broad range of biological activities, and are used in synthesis, catalysis and the design of materials (Gurbanov *et al.*, 2017 ▸, 2018 ▸; Mahmoudi *et al.*, 2018**a* ▸,*b* ▸,c* ▸; Mamedov *et al.*, 2018 ▸). Nowadays, N-ligands are key players in a wide diversity of fields, namely in coordination, metal–organic, pharmaceutical and medicinal chemistry, biologically active compounds, catalysis, non-covalent inter­actions and supra­molecular assemblies (Maharramov *et al.*, 2011 ▸, 2018 ▸; Mahmudov *et al.*, 2013 ▸, 2014 ▸, 2017**a* ▸,b* ▸, 2019 ▸; Mamedov *et al.*, 2015 ▸). In our previous studies we have reported on the mol­ecular structural properties of a series of 5-phenyl­thia­zolidin-2-imine derivatives (Akkurt *et al.*, 2018*a* ▸,*b* ▸; Duruskari *et al.*, 2019*a* ▸,*b* ▸; Khalilov *et al.*, 2019 ▸; Maharramov *et al.*, 2019 ▸). Following further study in this field, herein we report the crystal structure and Hirshfeld surface analysis of the title compound, (*E*)-3-(benzyl­idene­amino)-5-phenyl­thia­zolidin-2-iminium bromide.
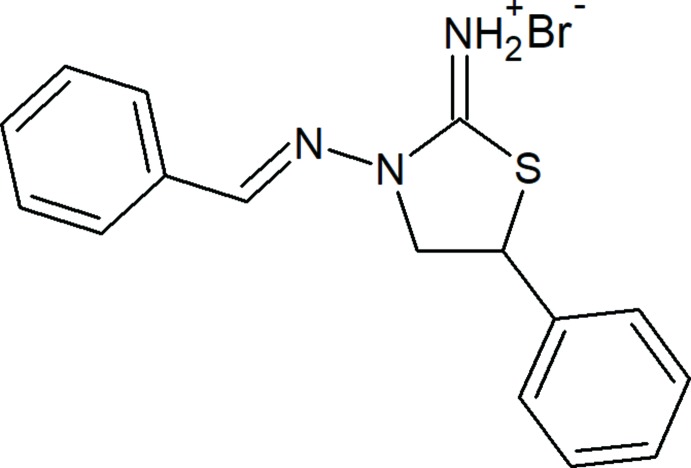


## Structural commentary   

**Figure 1:**
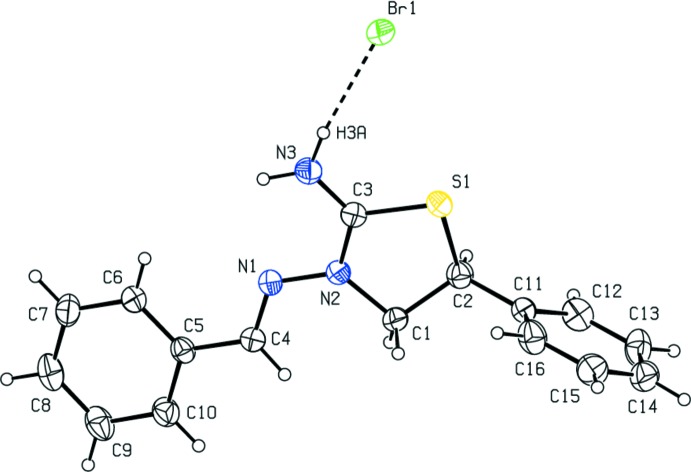
The mol­ecular structure of the title salt, with the atom labelling. Displacement ellipsoids are drawn at the 30% probability level. The inter­ionic hydrogen bond is shown as a dashed line.

The thia­zolidine ring (S1/N2/C1–C3) in the cation of the title salt (Fig. 1 ▸) adopts an envelope conformation, with the C atom bearing the phenyl ring as the flap atom; the puckering parameters are *Q*(2) = 0.318 (3) Å and *φ*(2) = 42.0 (5)°. The mean plane of the thia­zolidine ring makes dihedral angles of 18.28 (15) and 83.19 (15)°, respectively, with the C5–C10 and C11–C16 phenyl rings of the 3-(benzyl­idene­amino) and 5-phenyl­thia­zolidin groups, while the dihedral angle between them is 82.54 (15)°. The torsion angle of the N2—N1—C4—C5 bridge that links the thia­zolidine and 3-(benzyl­idene­amino) units is −175.7 (3)°.

## Supra­molecular features   

**Figure 2:**
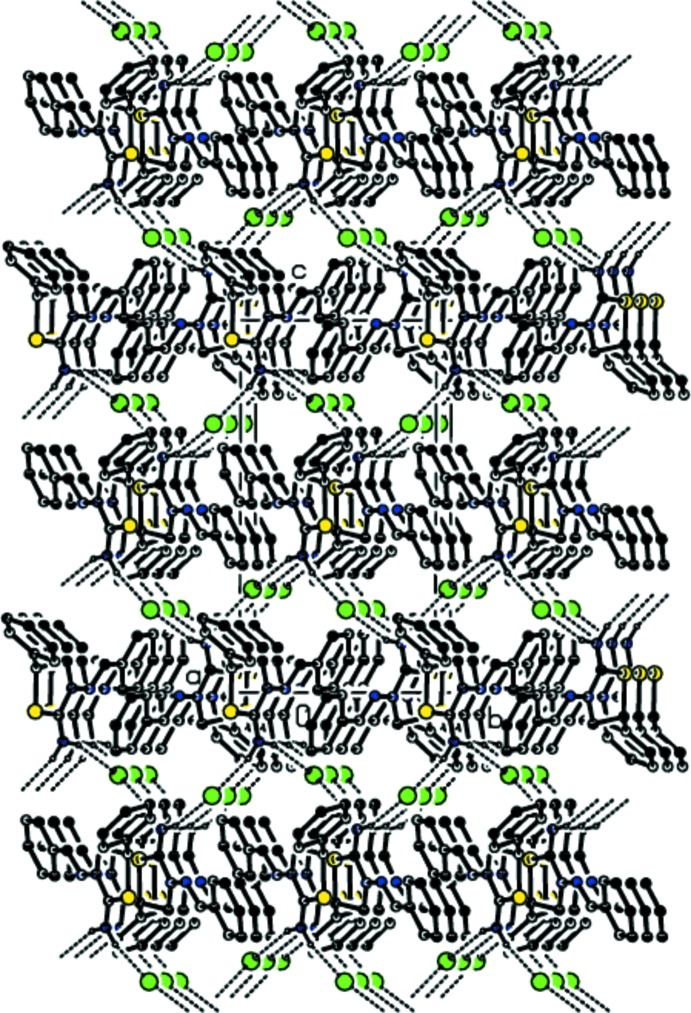
A view of the crystal packing showing the formation of chains parallel to the *b* axis through N—H⋯Br hydrogen bonds (dashed lines).

In the crystal, adjacent cations and anions are linked by pairs of N—H⋯Br hydrogen bonds (Table 1 ▸, Fig. 2 ▸), forming chains running parallel to the *b*-axis direction. C—H⋯π inter­actions or π–π stacking inter­actions contributing to the stabilization of the crystal packing are not observed.

## Hirshfeld surface analysis   

**Figure 3:**
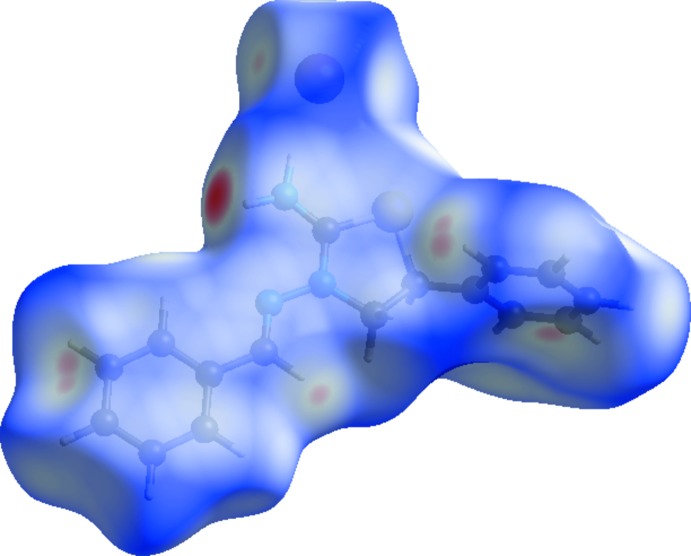
View of the three-dimensional Hirshfeld surface of the title salt plotted over *d*_norm_.

The Hirshfeld surface analysis (Spackman & Jayatilaka, 2009 ▸) of the title compound was generated by *CrystalExplorer 3.1* (Wolff *et al.*, 2012 ▸), and comprises *d_norm_* surface plots and two-dimensional fingerprint plots (Spackman & McKinnon, 2002 ▸). A *d*_norm_ surface plot of the title compound mapped over *d_norm_* using a standard surface resolution with a fixed colour scale of −0.3485 (red) to 1.3503 a.u. (blue) is shown in Fig. 3 ▸. The dark-red spots on the *d*_norm_ surface arise as a result of short inter­atomic contacts (Table 2 ▸), while the other weaker inter­molecular inter­actions appear as light-red spots.

**Figure 4:**
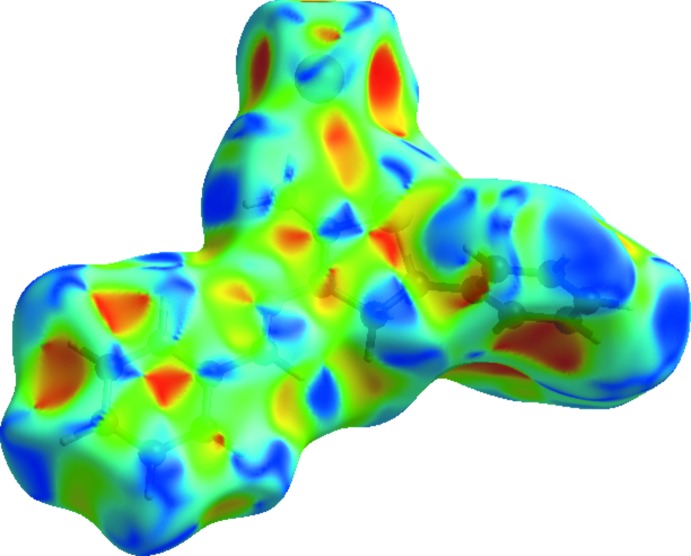
Hirshfeld surface of the title salt plotted over shape-index.

**Figure 5:**
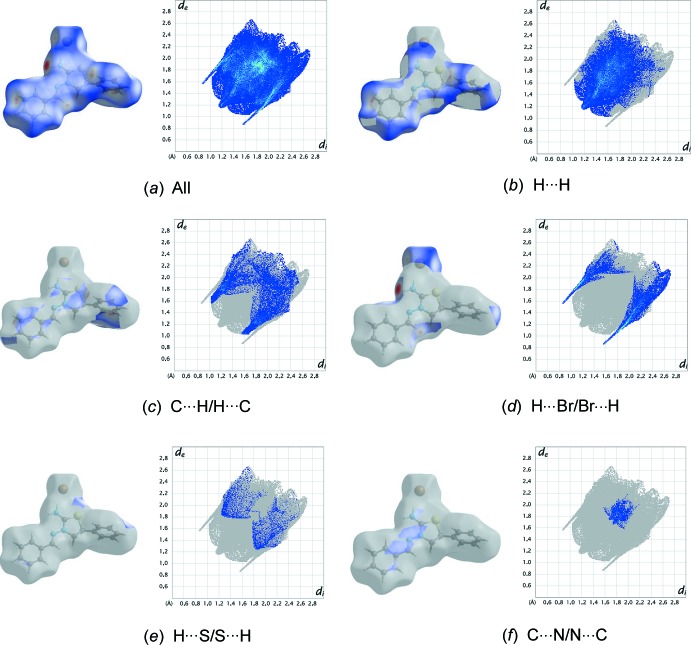
The Hirshfeld surface representations and the full two-dimensional fingerprint plots for the title salt, showing (*a*) all inter­actions, and delineated into (*b*) H⋯H, (*c*) C⋯H/H⋯C, (*d*) H⋯Br/Br⋯H, (*e*) H⋯S/S⋯H and (*f*) C⋯N/N⋯C inter­actions. The *d*_i_ and *d*_e_ values are the closest inter­nal and external distances (in Å) from given points on the Hirshfeld surface.

The shape index of the Hirshfeld surface is a tool to visualize π–·π stacking inter­actions by the presence of adjacent red and blue triangles; if there are no adjacent red and/or blue triangles, then there are no π–π inter­actions. Fig. 4 ▸ clearly suggests that there are no π–π inter­actions present in the title compound. Fig. 5 ▸(*a*) shows the two-dimensional fingerprint of the sum of the contacts contributing to the Hirshfeld surface represented in normal mode (Tables 1 ▸ and 2 ▸). The fingerprint plots delineated into H⋯H (46.4%), C⋯H/H⋯C (18.6%), H⋯Br/Br⋯H (17.5%), H⋯S/S⋯H (4.5%) and C⋯N/N⋯C (3.7%) contacts are shown in Fig. 5 ▸ *b*–*f*.

The most significant inter­molecular inter­actions are the H⋯H inter­actions (46.4%) (Fig. 5 ▸ *b*). All of the contributions to the Hirshfeld surface are given in Table 3 ▸. The large number of H⋯H, C⋯H/H⋯C and H⋯Br/Br⋯H inter­actions suggest that van der Waals inter­actions and hydrogen bonding play the major roles in the crystal packing (Hathwar *et al.*, 2015 ▸).

## Database survey   

A search of the Cambridge Structural Database (CSD, Version 5.40, February 2019; Groom *et al.*, 2016 ▸) for 2-thia­zolidiniminium compounds gave ten hits, *viz.* MOJGUQ (Duruskari *et al.*, 2019*a* ▸), XOWXAL (Duruskari *et al.*, 2019*b* ▸), BOBWIB (Khalilov *et al.*, 2019 ▸), UDELUN (Akkurt *et al.*, 2018*a* ▸), WILBIC (Marthi *et al.*, 1994 ▸), WILBOI (Marthi *et al.*, 1994 ▸), WILBOI01 (Marthi *et al.*, 1994 ▸), YITCEJ (Martem’yanova *et al.*, 1993*a* ▸), YITCAF (Martem’yanova *et al.*, 1993*b* ▸) and YOPLUK (Marthi *et al.*, 1995 ▸).

In the crystal of MOJGUQ (Duruskari *et al.*, 2019*a* ▸), centrosymmetrically related cations and anions are linked into dimeric units *via* N—-H⋯Br hydrogen bonds, which are further connected by weak C—H⋯Br contacts into chains parallel to the *a* axis. Furthermore, C—H⋯π inter­actions and π–π stacking inter­actions [centroid-to-centroid distance = 3.897 (2) Å] between the major components of the disordered phenyl ring contribute to the stabilization of the mol­ecular packing. In the crystal of XOWXAL (Duruskari *et al.*, 2019*b* ▸), the thia­zolidine ring adopts an envelope conformation. N—H⋯Br hydrogen bonds link the components into a three-dimensional network. Weak π–π stacking inter­actions between the phenyl rings of adjacent cations also contribute to the mol­ecular packing. In the crystal of BOBWIB (Khalilov *et al.*, 2019 ▸), the central thia­zolidine ring adopts an envelope conformation. In the crystal, centrosymmetrically related cations and anions are linked into dimeric units *via* N—H⋯Br hydrogen bonds, which are further connected by weak C—H⋯Br hydrogen bonds into chains parallel to [110]. In the crystal of UDELUN (Akkurt *et al.*, 2018*a* ▸), C—H⋯Br and N—H⋯Br hydrogen bonds link the components into a three-dimensional network with the cations and anions stacked along the *b*-axis direction. Weak C—H⋯π inter­actions, which only involve the minor disorder component of the ring, also contribute to the mol­ecular packing. In addition, there are also inversion-related Cl⋯Cl halogen bonds and C*-*–Cl⋯π(ring) contacts. In the other structures, the 3-N atom carries a C–substituent instead of an N–substituent as found in the title compound. Three of them were determined to be racemic (WILBIC; Marthi *et al.*, 1994 ▸) and two optically active samples (WILBOI and WILBOI01; Marthi *et al.*, 1994 ▸) of 3-(2′-chloro-2′-phenyl­eth­yl)-2-thia­zolidiniminium *p*-toluene­sulfonate. In all three structures, the most disordered fragment is the asymmetric C atom and the Cl atom attached to it. The disorder of the cation in the racemate corresponds to the presence of both enanti­omers at each site in the ratio 0.821 (3):0.179 (3). The system of hydrogen bonds connecting two cations and two anions into 12-membered rings is identical in the racemic and in the optically active crystals. YITCEJ (Martem’yanova *et al.*, 1993*a* ▸), is a product of the inter­action of 2-amino-5-methyl­thia­zoline with methyl iodide, with alkyl­ation at the endocylic nitro­gen atom, while YITCAF (Martem’yanova *et al.*, 1993*b* ▸) is a product of the reaction of 3-nitro-5-meth­oxy-, 3-nitro-5-chloro-, and 3-bromo-5-nitro­salicyl­aldehyde with the heterocyclic base to form the salt-like complexes.

## Synthesis and crystallization   

To the solution of 3-amino-5-phenyl­thia­zolidin-2-iminium bromide (1 mmol) in 20 mL of ethanol was added benzaldehyde (1 mmol) and the mixture was refluxed for 2 h. After cooling down to room temperature, the reaction product precipitated as colourless single crystals, which were collected by filtration and washed with cold acetone (yield 76%), m.p. 519 K. Analysis calculated for C_16_H_16_BrN_3_S (*M*_r_ = 362.29): C, 53.04; H, 4.45; N, 11.60. Found: C, 53.01; H, 4.42; N, 11.56%. ^1^H NMR (300 MHz, DMSO-*d*_6_) : 4.58 (*k*, 1H, CH_2_, ^3^ *J*_H–H_ = 6.9); 4,89 (*t*, 1H, CH_2_, ^3^ *J*_H–H_ =8.1); 5.60 (*t*, 1H, CH-Ar, ^3^ *J*_H–H_ =7.5); 7.37–8.07 (*m*, 10H, 10Ar-H); 8.44 (*s*, 1H, CH=), 10.35 (*s*, 2H, NH=). ^13^C NMR (75 MHz, DMSO-*d*_6_): 45.36, 55.91, 127.76, 128.65, 128.82, 128.86, 129.09, 131.54, 132.85, 137.48, 151.11, 167.84. MS (ESI), *m*/*z*: 282.30 [C_16_H_16_N_3_S]^+^ and 79.88 Br^−^.

## Refinement   

Crystal data, data collection and structure refinement details are summarized in Table 4 ▸. All H atoms were placed at calculated positions (N—H = 0.90 Å and C—H = 0.93–0.98 Å) and refined using a riding model, with *U*_iso_(H) = 1.2*U*_eq_(N, C). The distances between the carbon atoms of two phenyl groups were constrained with a DFIX instruction [DFIX 1.40 0.02 C C].

## Supplementary Material

Crystal structure: contains datablock(s) I. DOI: 10.1107/S2056989020001899/rz5269sup1.cif

Structure factors: contains datablock(s) I. DOI: 10.1107/S2056989020001899/rz5269Isup2.hkl

CCDC reference: 1837123

Additional supporting information:  crystallographic information; 3D view; checkCIF report

## Figures and Tables

**Table 1:** Hydrogen-bond geometry (Å, °)

*D*—H⋯*A*	*D*—H	H⋯*A*	*D*⋯*A*	*D*—H⋯*A*
N3—H3*A*⋯Br1	0.90	2.37	3.258 (3)	168
N3—H3*B*⋯Br1^i^	0.90	2.55	3.399 (3)	158

Symmetry code: (i) 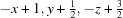.

**Table 2:** Summary of short inter­atomic contacts (Å) in the title salt

Contact	Distance	Symmetry operation
Br1⋯H3*A* (N3)	2.37	*x*, *y*, *z*
Br1⋯H3*B* (N3)	2.55	1 − *x*, − 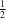 + *y*, 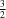 − *z*
Br1⋯H14*A* (C14)	3.14	−*x*, 1 − *y*, 1 − *z*
Br1⋯H4*A* (C4)	2.96	*x*, 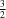 − *y*, 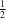 + *z*
Br1⋯H12*A* (C12)	3.02	*x*, 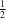 − *y*, 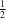 + *z*

**Table 3:** Percentage contributions of inter­atomic contacts to the Hirshfeld surface for the title salt

Contact	Percentage contribution
H⋯H	46.4
C⋯H/H⋯C	18.6
H⋯Br/Br⋯H	17.5
H⋯S/S⋯H	4.5
C⋯N/N⋯C	3.7
C⋯S/S⋯C	3.0
H⋯N/N⋯H	2.6
C⋯C	2.3
C⋯Br/Br⋯C	0.9
N⋯S/S⋯N	0.5
N⋯N	0.2

**Table 4:** Experimental details

Crystal data	
Chemical formula	C_16_H_16_N_3_S^+^·Br^−^
*M*_r_	362.29
Crystal system, space group	Monoclinic, *P*2_1_/*c*
Temperature (K)	296
*a*, *b*, *c* (Å)	12.138 (8), 8.336 (5), 15.872 (9)
β (°)	93.910 (16)
*V* (Å^3^)	1602.3 (17)
*Z*	4
Radiation type	Mo *K*α
μ (mm^−1^)	2.69
Crystal size (mm)	0.21 × 0.18 × 0.13

Data collection	
Diffractometer	Bruker APEXII CCD
Absorption correction	Multi-scan (*SADABS*; Bruker, 2003 ▸)
*T*_min_, *T*_max_	0.582, 0.713
No. of measured, independent and observed [*I* > 2σ(*I*)] reflections	23979, 3314, 2742
*R*_int_	0.049
(sin θ/λ)_max_ (Å^−1^)	0.629

Refinement	
*R*[*F* ^2^ > 2σ(*F* ^2^)], *wR*(*F* ^2^), *S*	0.040, 0.111, 1.06
No. of reflections	3314
No. of parameters	190
No. of restraints	12
H-atom treatment	H-atom parameters constrained
Δρ_max_, Δρ_min_ (e Å^−3^)	0.74, −0.60

Computer programs: *APEX2* and *SAINT* (Bruker, 2003 ▸), *SHELXT2014* (Sheldrick, 2015*a* ▸), *SHELXL2016* (Sheldrick, 2015*b* ▸) and *SHELXTL* (Sheldrick, 2008 ▸).

## supplementary crystallographic information

### Crystal data

**Table:**

C_16_H_16_N_3_S^+^·Br^−^	*F*(000) = 736
*M**_r_* = 362.29	*D*_x_ = 1.502 Mg m^−^^3^
Monoclinic, *P*2_1_/*c*	Mo *K*α radiation, λ = 0.71073 Å
*a* = 12.138 (8) Å	Cell parameters from 9891 reflections
*b* = 8.336 (5) Å	θ = 2.8–26.4°
*c* = 15.872 (9) Å	µ = 2.69 mm^−^^1^
β = 93.910 (16)°	*T* = 296 K
*V* = 1602.3 (17) Å^3^	Plate, colourless
*Z* = 4	0.21 × 0.18 × 0.13 mm

### Data collection

**Table:**

Bruker APEXII CCD diffractometer	2742 reflections with *I* > 2σ(*I*)
φ and ω scans	*R*_int_ = 0.049
Absorption correction: multi-scan (SADABS; Bruker, 2003)	θ_max_ = 26.5°, θ_min_ = 2.6°
*T*_min_ = 0.582, *T*_max_ = 0.713	*h* = −15→15
23979 measured reflections	*k* = −10→10
3314 independent reflections	*l* = −19→18

### Refinement

**Table:**

Refinement on *F*^2^	Primary atom site location: structure-invariant direct methods
Least-squares matrix: full	Secondary atom site location: difference Fourier map
*R*[*F*^2^ > 2σ(*F*^2^)] = 0.040	Hydrogen site location: mixed
*wR*(*F*^2^) = 0.111	H-atom parameters constrained
*S* = 1.06	*w* = 1/[σ^2^(*F*_o_^2^) + (0.0521*P*)^2^ + 1.4845*P*] where *P* = (*F*_o_^2^ + 2*F*_c_^2^)/3
3314 reflections	(Δ/σ)_max_ < 0.001
190 parameters	Δρ_max_ = 0.74 e Å^−^^3^
12 restraints	Δρ_min_ = −0.60 e Å^−^^3^

### Special details

**Table:**

Geometry. All esds (except the esd in the dihedral angle between two l.s. planes) are estimated using the full covariance matrix. The cell esds are taken into account individually in the estimation of esds in distances, angles and torsion angles; correlations between esds in cell parameters are only used when they are defined by crystal symmetry. An approximate (isotropic) treatment of cell esds is used for estimating esds involving l.s. planes.

### Fractional atomic coordinates and isotropic or equivalent isotropic displacement parameters (Å^2^)

**Table:**

	*x*	*y*	*z*	*U*_iso_*/*U*_eq_	
Br1	0.36371 (3)	0.40930 (5)	0.77766 (2)	0.05996 (15)	
S1	0.27771 (6)	0.50993 (10)	0.55109 (5)	0.0478 (2)	
N1	0.52247 (19)	0.7685 (3)	0.49030 (15)	0.0411 (5)	
N2	0.42403 (19)	0.6853 (3)	0.48814 (15)	0.0438 (5)	
N3	0.4551 (2)	0.6390 (3)	0.63168 (16)	0.0500 (6)	
H3A	0.431514	0.589059	0.677388	0.060*	
H3B	0.509254	0.711959	0.640888	0.060*	
C1	0.3432 (2)	0.6653 (4)	0.41558 (19)	0.0467 (7)	
H1A	0.380449	0.651339	0.363896	0.056*	
H1B	0.295261	0.758163	0.409430	0.056*	
C2	0.2763 (3)	0.5140 (4)	0.4348 (2)	0.0489 (7)	
H2A	0.315568	0.419380	0.415778	0.059*	
C3	0.3971 (2)	0.6205 (3)	0.56063 (18)	0.0412 (6)	
C4	0.5460 (2)	0.8450 (4)	0.42487 (19)	0.0440 (6)	
H4A	0.495374	0.850348	0.378264	0.053*	
C5	0.6520 (2)	0.9241 (3)	0.42299 (14)	0.0416 (6)	
C6	0.7284 (2)	0.9221 (4)	0.49283 (19)	0.0513 (7)	
H6A	0.710541	0.874346	0.543049	0.062*	
C7	0.8319 (2)	0.9924 (4)	0.4867 (2)	0.0663 (10)	
H7A	0.883751	0.988871	0.532539	0.080*	
C8	0.8581 (3)	1.0679 (4)	0.41226 (19)	0.0652 (10)	
H8A	0.927072	1.114885	0.408687	0.078*	
C9	0.7808 (2)	1.0730 (4)	0.3432 (2)	0.0651 (10)	
H9A	0.797532	1.124866	0.293831	0.078*	
C10	0.6782 (2)	0.9998 (4)	0.34862 (17)	0.0551 (8)	
H10A	0.626928	1.001586	0.302329	0.066*	
C11	0.1609 (2)	0.5115 (3)	0.39571 (17)	0.0446 (6)	
C12	0.1299 (2)	0.3891 (4)	0.3387 (2)	0.0613 (9)	
H12A	0.180793	0.310914	0.325946	0.074*	
C13	0.0225 (2)	0.3838 (5)	0.3009 (2)	0.0690 (10)	
H13A	0.002162	0.302592	0.262799	0.083*	
C14	−0.0538 (3)	0.5001 (4)	0.3205 (2)	0.0661 (10)	
H14A	−0.124515	0.498545	0.293897	0.079*	
C15	−0.0251 (3)	0.6186 (4)	0.3794 (2)	0.0683 (10)	
H15A	−0.076995	0.693888	0.394077	0.082*	
C16	0.0820 (2)	0.6235 (4)	0.4163 (2)	0.0631 (9)	
H16A	0.101386	0.703134	0.455623	0.076*	

### Atomic displacement parameters (Å^2^)

**Table:**

	*U*^11^	*U*^22^	*U*^33^	*U*^12^	*U*^13^	*U*^23^
Br1	0.0594 (2)	0.0700 (3)	0.0489 (2)	−0.01088 (16)	−0.00776 (15)	0.01409 (15)
S1	0.0461 (4)	0.0511 (4)	0.0467 (4)	−0.0073 (3)	0.0065 (3)	0.0068 (3)
N1	0.0379 (12)	0.0421 (13)	0.0432 (13)	−0.0028 (10)	0.0028 (10)	0.0008 (10)
N2	0.0387 (12)	0.0496 (14)	0.0429 (13)	−0.0054 (10)	0.0013 (10)	0.0085 (11)
N3	0.0544 (15)	0.0552 (15)	0.0405 (13)	−0.0076 (12)	0.0042 (11)	0.0037 (11)
C1	0.0426 (15)	0.0550 (17)	0.0418 (15)	−0.0053 (13)	−0.0020 (12)	0.0096 (13)
C2	0.0471 (16)	0.0482 (17)	0.0515 (17)	−0.0002 (13)	0.0038 (13)	−0.0012 (14)
C3	0.0406 (14)	0.0414 (14)	0.0419 (15)	0.0029 (11)	0.0062 (12)	0.0012 (12)
C4	0.0436 (15)	0.0431 (15)	0.0449 (15)	−0.0018 (12)	0.0005 (12)	0.0055 (12)
C5	0.0422 (15)	0.0334 (14)	0.0497 (16)	−0.0004 (11)	0.0063 (12)	−0.0022 (12)
C6	0.0536 (18)	0.0424 (16)	0.0566 (18)	−0.0077 (13)	−0.0055 (15)	0.0040 (14)
C7	0.053 (2)	0.055 (2)	0.087 (3)	−0.0103 (16)	−0.0162 (18)	−0.0012 (19)
C8	0.0486 (19)	0.059 (2)	0.089 (3)	−0.0127 (16)	0.0169 (19)	−0.0111 (19)
C9	0.070 (2)	0.068 (2)	0.059 (2)	−0.0205 (19)	0.0244 (18)	−0.0054 (17)
C10	0.059 (2)	0.060 (2)	0.0468 (17)	−0.0132 (16)	0.0068 (15)	−0.0005 (15)
C11	0.0407 (15)	0.0474 (16)	0.0457 (15)	−0.0051 (12)	0.0039 (12)	0.0059 (13)
C12	0.061 (2)	0.059 (2)	0.065 (2)	0.0023 (16)	0.0165 (17)	−0.0059 (17)
C13	0.063 (2)	0.082 (3)	0.061 (2)	−0.022 (2)	0.0058 (18)	−0.0193 (19)
C14	0.054 (2)	0.083 (3)	0.061 (2)	−0.0111 (19)	0.0019 (17)	0.010 (2)
C15	0.055 (2)	0.062 (2)	0.088 (3)	0.0063 (17)	0.0043 (19)	0.007 (2)
C16	0.060 (2)	0.0477 (18)	0.083 (3)	−0.0009 (15)	0.0096 (18)	−0.0116 (17)

### Geometric parameters (Å, º)

**Table:**

S1—C3	1.716 (3)	C6—H6A	0.9300
S1—C2	1.844 (3)	C7—C8	1.394 (2)
N1—C4	1.268 (4)	C7—H7A	0.9300
N1—N2	1.380 (3)	C8—C9	1.394 (2)
N2—C3	1.332 (4)	C8—H8A	0.9300
N2—C1	1.470 (4)	C9—C10	1.394 (2)
N3—C3	1.297 (4)	C9—H9A	0.9300
N3—H3A	0.9000	C10—H10A	0.9300
N3—H3B	0.9001	C11—C16	1.392 (2)
C1—C2	1.542 (4)	C11—C12	1.398 (2)
C1—H1A	0.9700	C12—C13	1.397 (2)
C1—H1B	0.9700	C12—H12A	0.9300
C2—C11	1.493 (4)	C13—C14	1.391 (2)
C2—H2A	0.9800	C13—H13A	0.9300
C4—C5	1.447 (4)	C14—C15	1.389 (2)
C4—H4A	0.9300	C14—H14A	0.9300
C5—C10	1.3944 (19)	C15—C16	1.390 (2)
C5—C6	1.396 (2)	C15—H15A	0.9300
C6—C7	1.395 (2)	C16—H16A	0.9300
			
C3—S1—C2	91.65 (14)	C5—C6—H6A	120.3
C4—N1—N2	118.4 (2)	C8—C7—C6	120.5 (3)
C3—N2—N1	116.4 (2)	C8—C7—H7A	119.8
C3—N2—C1	116.2 (2)	C6—C7—H7A	119.8
N1—N2—C1	127.4 (2)	C9—C8—C7	120.0 (3)
C3—N3—H3A	117.5	C9—C8—H8A	120.0
C3—N3—H3B	124.6	C7—C8—H8A	120.0
H3A—N3—H3B	116.8	C8—C9—C10	119.6 (3)
N2—C1—C2	105.7 (2)	C8—C9—H9A	120.2
N2—C1—H1A	110.6	C10—C9—H9A	120.2
C2—C1—H1A	110.6	C9—C10—C5	120.4 (3)
N2—C1—H1B	110.6	C9—C10—H10A	119.8
C2—C1—H1B	110.6	C5—C10—H10A	119.8
H1A—C1—H1B	108.7	C16—C11—C12	118.8 (3)
C11—C2—C1	114.9 (3)	C16—C11—C2	122.2 (2)
C11—C2—S1	111.1 (2)	C12—C11—C2	118.9 (2)
C1—C2—S1	104.1 (2)	C13—C12—C11	120.2 (3)
C11—C2—H2A	108.8	C13—C12—H12A	119.9
C1—C2—H2A	108.8	C11—C12—H12A	119.9
S1—C2—H2A	108.8	C14—C13—C12	119.9 (3)
N3—C3—N2	123.5 (3)	C14—C13—H13A	120.1
N3—C3—S1	123.1 (2)	C12—C13—H13A	120.1
N2—C3—S1	113.4 (2)	C15—C14—C13	120.4 (3)
N1—C4—C5	119.7 (3)	C15—C14—H14A	119.8
N1—C4—H4A	120.1	C13—C14—H14A	119.8
C5—C4—H4A	120.1	C14—C15—C16	119.3 (3)
C10—C5—C6	120.0 (3)	C14—C15—H15A	120.3
C10—C5—C4	118.6 (2)	C16—C15—H15A	120.3
C6—C5—C4	121.4 (2)	C15—C16—C11	121.3 (3)
C7—C6—C5	119.5 (3)	C15—C16—H16A	119.3
C7—C6—H6A	120.3	C11—C16—H16A	119.3
			
C4—N1—N2—C3	−173.3 (3)	C5—C6—C7—C8	1.7 (5)
C4—N1—N2—C1	4.2 (4)	C6—C7—C8—C9	−0.3 (6)
C3—N2—C1—C2	−24.6 (4)	C7—C8—C9—C10	−1.1 (6)
N1—N2—C1—C2	157.9 (3)	C8—C9—C10—C5	1.0 (6)
N2—C1—C2—C11	151.9 (3)	C6—C5—C10—C9	0.4 (5)
N2—C1—C2—S1	30.1 (3)	C4—C5—C10—C9	−177.9 (3)
C3—S1—C2—C11	−148.7 (2)	C1—C2—C11—C16	−63.2 (4)
C3—S1—C2—C1	−24.5 (2)	S1—C2—C11—C16	54.7 (4)
N1—N2—C3—N3	3.6 (4)	C1—C2—C11—C12	119.2 (3)
C1—N2—C3—N3	−174.1 (3)	S1—C2—C11—C12	−122.9 (3)
N1—N2—C3—S1	−176.5 (2)	C16—C11—C12—C13	2.5 (5)
C1—N2—C3—S1	5.8 (3)	C2—C11—C12—C13	−179.9 (3)
C2—S1—C3—N3	−168.0 (3)	C11—C12—C13—C14	−0.4 (6)
C2—S1—C3—N2	12.1 (2)	C12—C13—C14—C15	−2.1 (6)
N2—N1—C4—C5	−175.7 (3)	C13—C14—C15—C16	2.5 (6)
N1—C4—C5—C10	176.5 (3)	C14—C15—C16—C11	−0.3 (6)
N1—C4—C5—C6	−1.9 (5)	C12—C11—C16—C15	−2.1 (6)
C10—C5—C6—C7	−1.8 (5)	C2—C11—C16—C15	−179.7 (3)
C4—C5—C6—C7	176.5 (3)		

### Hydrogen-bond geometry (Å, º)

**Table:**

*D*—H···*A*	*D*—H	H···*A*	*D*···*A*	*D*—H···*A*
N3—H3*A*···Br1	0.90	2.37	3.258 (3)	168
N3—H3*B*···Br1^i^	0.90	2.55	3.399 (3)	158

Symmetry code: (i) −*x*+1, *y*+1/2, −*z*+3/2.
